# A cluster randomized trial of a Multicomponent Clinical Care Pathway (MCCP) to improve MASLD diagnosis and management in primary care: study protocol

**DOI:** 10.1186/s12913-025-12737-2

**Published:** 2025-05-06

**Authors:** Kyler M. Godwin, Larissa Grigoryan, Aaron P. Thrift, Hao Duong, Fasiha Kanwal, Traber Giardina, Himabindu Kadiyala, Andrew Zimolzak, Kavish R. Patidar, Hashem B. El-Serag

**Affiliations:** 1 Department of Medicine, Section of Health Services Research, Baylor College of Medicine, Houston, TX USA; 2 Center for Innovations in Quality, Effectiveness and Safety (IQuESt), Michael E. DeBakey Veterans Affairs Medical Center, 2002 Holcombe Blvd. (152), Houston, TX 77030 USA; 3 Department of Family and Community Medicine, Baylor College of Medicine, Houston, TX USA; 4 Section of Epidemiology and Population Sciences, Department of Medicine, Baylor College of Medicine, Houston, TX USA; 5 Dan L Duncan Comprehensive Cancer Center, Baylor College of Medicine, Houston, TX USA; 6 Section of Gastroenterology and Hepatology, Department of Medicine, Baylor College of Medicine, Houston, TX USA; 7 Section of General Internal Medicine, Department of Medicine, Baylor College of Medicine, Houston, TX USA

**Keywords:** Fatty liver, Veterans, Primary Care, Implementation Science, Predictive Algorithm, FIB- 4, Metabolic syndrome, Metabolic dysfunction associated steatohepatitis, MASH

## Abstract

**Background:**

Metabolic dysfunction associated steatotic liver disease (MASLD) is the most common chronic liver disorder in the world. Most patients with MASLD are undiagnosed, untreated and unreferred. Treatment depends on diagnosis and accurate staging of fibrosis risk, and therefore screening at the primary care setting coupled with consistent, timely, evidence-based, widely accessible, and testable management processes is critical. Randomized controlled trials of methods for screening, diagnosis, severity stratification, and referral are lacking. We previously validated the MASLD clinical care pathway (MCCP), a multistep algorithmic process geared to support primary care settings in screening, diagnosis, risk stratification, and suggested referral and treatment of patients with MASLD. We will evaluate the effectiveness of the MCCP intervention compared to usual care in improving MASLD care process and patient outcomes.

**Methods:**

A two-arm, parallel, cluster-randomized trial will assess the effect of the MCCP intervention comprised of an e-trigger and structured provider education on screening, diagnosis, referral, and treatment for MASLD. Clusters are Veterans Affairs (VA) patient-centered primary care clinics, called Patient Aligned Care Teams (PACTs). This 4-year study will include three phases: (1) formative assessment to explore barriers to feasibility and acceptability of intervention among providers and patients and adapt MCCP for utilization by VA PACTs, (2) cluster-randomized trial to examine the effectiveness of the MCCP compared to usual care, and (3) summative evaluation to identify patient and provider characteristics associated with effectiveness of MCCP and plan for future implementation. The primary outcome will be a composite dichotomous variable for making a new MASLD diagnosis and completing severity risk stratification of MASLD. Secondary outcomes include referral and receipt of hepatology specialty care for patients with MASLD and advanced fibrosis.

**Discussion:**

Our randomized controlled trial will be the first to systematically implement the MCCP using strategies that are clinically relevant, adaptable and scalable. If successful, the MCCP will improve outcomes for patients with MASLD through early detection, accurate risk stratification, and timely treatment within primary care settings.

**Trial registration:**

This trial has been registered on clinicaltrails.gov (# NCT06671886), registration date: 10–31-2024.

**Supplementary Information:**

The online version contains supplementary material available at 10.1186/s12913-025-12737-2.

## Introduction

Metabolic dysfunction associated steatotic liver disease (MASLD), formerly known as nonalcoholic fatty liver disease (NAFLD), is characterized by an accumulation of excess fat in the liver consequent to metabolic syndrome and has become the most common chronic liver disease in the world [1–3]. In the United States, MASLD affects 25–40% of the general adult population [4–8]. Most people with MASLD have mild or non-progressive disease [7]. However, in approximately 30% of cases, MASLD progresses from quiescent simple steatosis to a necroinflammatory disease (metabolic dysfunction associated steatohepatitis (MASH) associated with progressive hepatic fibrosis [9, 10], which may lead to cirrhosis in a substantial proportion of patients [11]. Patients with advanced hepatic fibrosis are at greatest risk of developing complications of chronic liver disease, including hepatocellular carcinoma (HCC), increased risk of cardiovascular disease and overall increase in mortality [12, 13], and are therefore the most important subgroup to identify and most urgent to treat.

High-risk groups for MALSD and advanced fibrosis can be identified in clinical practice. Most patients with MASLD (> 90%) have one or more metabolic conditions (e.g., diabetes mellitus (DM), obesity, hypertension) that are also associated with a 2–threefold increased risk of advanced hepatic fibrosis. Screening guidelines focus on people with these features. Validated noninvasive scores incorporating commonly available demographic and clinical variables (e.g., FIB- 4) as well as ultrasound-based vibration controlled transient elastography (e.g., Fibroscan) are also widely available for MASLD risk stratification. Accurate identification and risk stratification of people with MASLD are likely to have positive consequences. For all patients with MASLD, practice guidelines (e.g., American Association for the Study of Liver Disease, European Association for the Study of the Liver) recommend weight loss in overweight or obese patients as well as education about nutritional strategies, regular physical activity, and avoiding excess alcohol intake. There is compelling evidence of short and intermediate benefits of weight loss on objective-relevant outcomes. For example, a dose–response relationship was found between weight loss and resolution of MASH in a meta-analysis of 43 studies, including 2809 individuals treated with structured weight loss programs, pharmacotherapy, or bariatric surgery [14]. For patients with MASLD and advanced fibrosis, referral to a hepatology service is recommended to monitor and manage cirrhosis and HCC, use specific medications or enroll in clinical trials. At present, there is one FDA-approved pharmacologic agent for treating MASH (resmetirom) [15], and several incretins have shown highly promising results in phase 2 trials [16].

Because MASLD is largely asymptomatic and treatment depends on accurate staging of fibrosis risk, most patients with MASLD in clinical practice are undetected, unmanaged, and unreferred [17, 18]. For example, we previously conducted a structured medical record review of 251 Veterans with MASLD and found that approximately 40% had documentation of abnormal ALT, only 21.5% had MASLD mentioned as a possible diagnosis, less than 15% were counseled regarding diet and exercise, and only 10.4% were referred to a specialist [17].

Screening at the primary care setting coupled with consistent, timely, evidence-based, widely accessible, and testable management processes is critical. However, there is no evidence-based guidance on optimal methods for screening, diagnosis, severity stratification, and referral. Several groups and professional societies [3, 19, 20] advocate the use of screeners combined with clinical care pathways. However, the effectiveness of the available guidelines has not been tested in a randomized controlled study.

We previously developed and validated an electronic (e-trigger) tool [7] aimed at the identification and risk stratification of patients at high risk for moderate to advanced fibrosis in primary care in the department of Veterans Affairs (VA) [7]. In this trial, we will test the effectiveness of an intervention that adapts this e-trigger as part of a clinical care pathway for use in VA primary care. This intervention could prevent MASLD-related complications, but prospective effectiveness data are required before wide implementation is recommended. The multicomponent MASLD clinical care pathway (MCCP) study will also provide key information about the determinants of feasibility, acceptability and implementation of multicomponent clinical care pathway intervention to improve MASLD diagnosis and management in primary care**.**

## Methods

### Study design

**Fig. 1:**
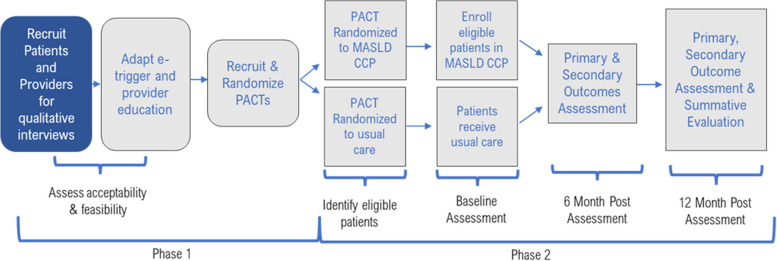
Study design

A two-arm, parallel, cluster-randomized trial will assess the effect of a MCCP intervention comprised of an e-trigger and structured provider education on the MASLD screening, diagnosis, referral, and treatment. Clusters are VA patient-centered primary care clinics, called Patient Aligned Care Teams (PACTs). The trial will randomize 16 PACTs in a 1:1 allocation ratio to two arms: MCCP intervention versus usual care. These 16 PACTs will be randomly selected from the 37 eligible PACTs, excluding those participated in focus groups. We will also conduct mixed-methods formative and summative evaluations of the intervention throughout the 4 years of the study. The study is designed with pragmatic features to foster efficient implementation of findings into clinical care settings. Figure 1 depicts the study design and procedures.

### Specific aims and hypotheses

The MCCP study is designed to test the hypothesis that the PACTs randomized to a multicomponent MCCP intervention will have increased guideline-concordant diagnosis, referral and treatment than the control PACTs. *Specific Aim 1* (formative assessment), which will be performed in year 1, is to assess barriers to feasibility and acceptability of the MCPP intervention among providers and patients and adapt MCCP for utilization by VA PACTs. *Specific Aim 2* (cluster-randomized trial), performed over years 2–4, is to examine the effectiveness of the MCCP compared to usual care in improving MASLD care process and patient outcomes. *Specific Aim 3* (summative evaluation)*,* performed in year 4, is to identify patient and provider characteristics associated with effectiveness of MCCP and plan for future implementation.

### Study setting

The study will take place at the Michael E. DeBakey VA Medical Center (MEDVAMC) in Houston, Texas, which provides care for more than 111,000 Veterans and has a total of 37 PACTs. The typical PACT core team consists of a primary care provider (physician, nurse practitioner, or physician assistant), a nurse care manager (registered nurse), a clinical associate (practice nurse or health technician), and a clerk. Teams work closely with multidisciplinary professionals including pharmacists, social workers, nutritionists, and specialist providers. The study and principal investigators will be introduced to the PACT’s lead primary care providers (PCP) during department meetings. Those who are interested will be recruited to advise on the feasibility and acceptability of the intervention.

Specialized PACTs such as infectious disease, spinal cord injury, homeless, and geriatrics are excluded due to patients facing more pressing issues. The Post-deployment PACT is excluded due to unique care needs and having only one team. We also exclude PACTs with fewer than 100 patients expected in the three-month period. Additionally, unstable PACT leadership (e.g., pending departure or vacancy) at the time of randomization will be excluded. The biostatistician will randomize PACTs into the intervention or usual care groups with a computer-generated randomization.

### Participants in specific aim 1

To understand the feasibility and acceptability of the e-trigger, the research team will identify a random sample of patients with already diagnosed MASLD and receiving care at MEDVAMC within the past year using electronic health record search and chart review. Phone call visits are excluded as they are typically brief, addressing lab results or medication changes. Potential participants will be contacted during a clinic encounter to determine interest in participating. Patients who agree to participate will be scheduled for a telephone/video interview. We will recruit patients on a rolling basis until we reach our sample goal of 20 patients or reach saturation in the themes patients are reporting. Six PACTs will be interviewed to understand the barriers and facilitators to implementing the e-trigger.

### Conceptual framework

**Fig. 2:**
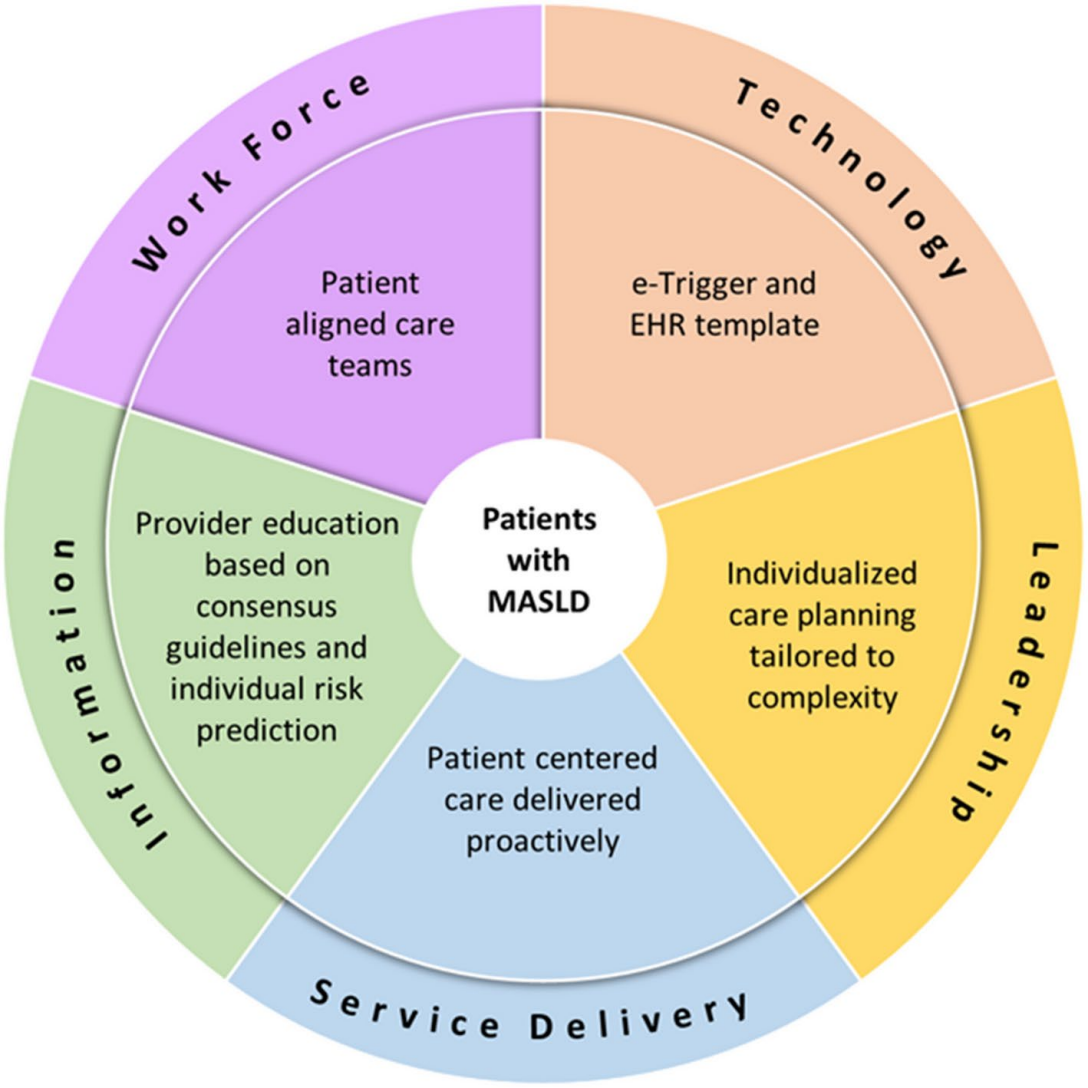
Conceptual model

Our multicomponent intervention design is grounded in the SELFIE (Sustainable intEgrated care modeLs for multi-morbidity: delivery, Financing and perfromanceE) Framework (Fig. 2) [21]. Care components cover the World Health Organization’s six dimensions of health systems: information, workforce, technologies, leadership, service delivery, and financing. Our interprofessional team developed the MCCP clinician education based upon consensus informed guidelines and individual risk prediction models (*Information*). The MCCP includes an interprofessional team of a provider and a nurse, at minimum, embedded within each PACT (*Workforce*). E-triggers, developed based on the Safer Dx Trigger Tool Framework [22], are used along with other clinician facing technologies such as note templates (*Technology*). Clinicians and their patients share responsibility for guiding individualized care planning that is tailored to a patient’s needs *(Leadership).* The MCCP provides recommendations on referral and treatment for MASLD patients to reduce gaps in care and proactively deliver patient-centered care (*Service delivery*). *Financing* (e.g., evaluation of cost-effectiveness) will be addressed in future studies after the MCCP effectiveness is established.

### Study phases and timeline

The study will be conducted in two phases over 4 years. The focus group questions will be aligned with the Consolidated Framework for Implementation Research (CFIR) 2.0 domains and constructs [23]. The second phase (years 2–4) is a cluster-randomized trial and summative evaluation of intervention effectiveness based on the Reach, Effectiveness, Adoption, Implementation and Maintenance (RE-AIM) measures to inform future implementation [24].

### Intervention

**Fig. 3:**
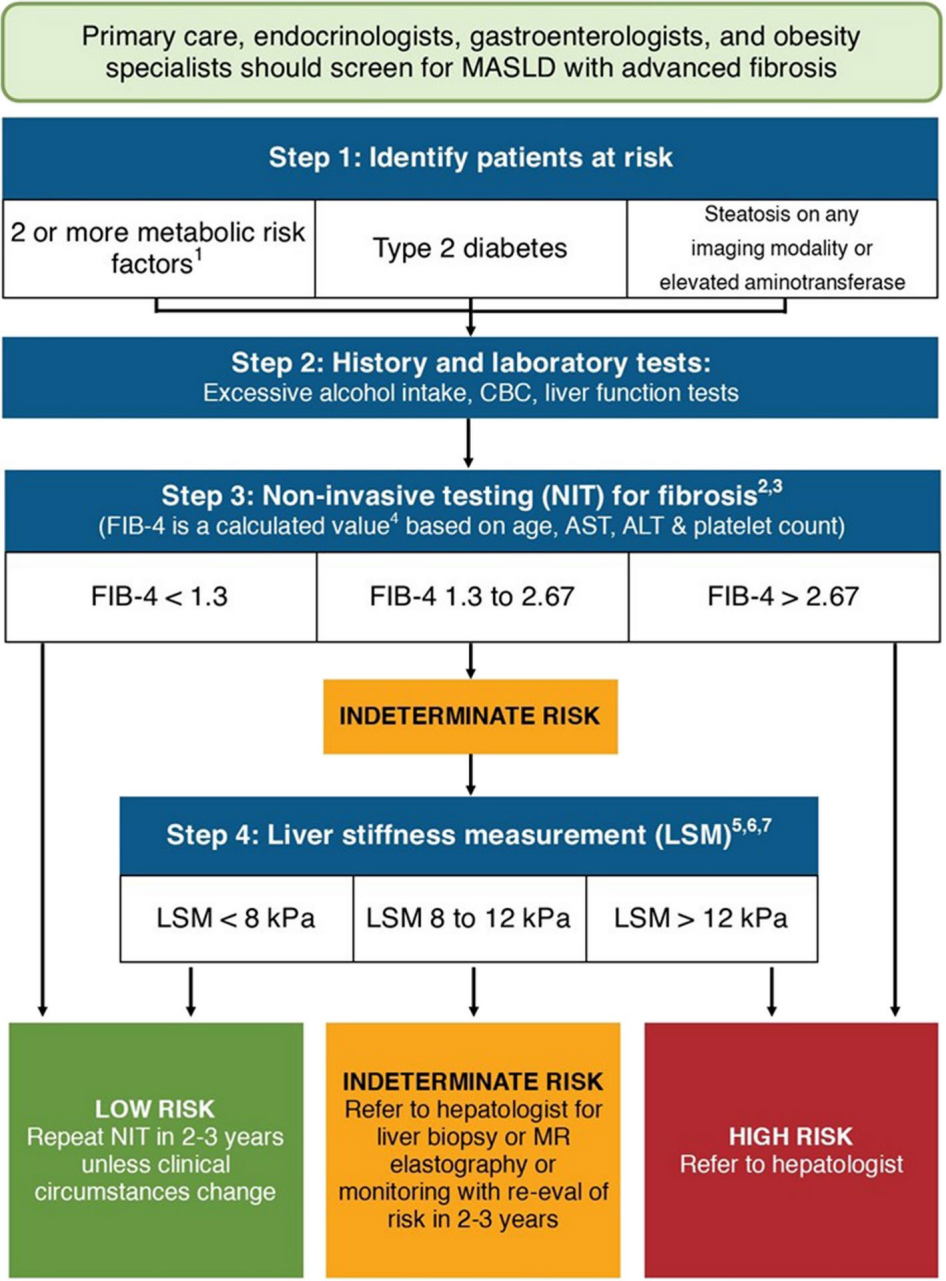
MASLD clinical care pathway

Our validated MCCP [7] includes algorithmic guidance geared towards primary care providers for the screening, diagnosis, risk stratification, referral and treatment for patients with MASLD (Fig. 3). This multistep pathway entails identification of patients at risk (Step 1), targeted history and lab testing (Step 2), noninvasive testing for hepatic fibrosis using FIB- 4 (Step 3), elective additional testing with Fibroscan for those with indeterminate FIB- 4 (Step 4), and recommended management and follow-up.

Our MASLD intervention is multicomponent. First, we will identify high-risk patients using the e-trigger algorithm that was previously developed and validated among randomly selected patients in primary care at MEDVAMC aimed at the identification of MASLD and initial risk stratification of patients in VA primary care [7]. Second, we will educate and train PACT personnel on how to deal with e-trigger positive patients as well as make treatment recommendations within an interprofessional team. The e-trigger is a logistic regression model that contains three predictive variables (BMI, FIB- 4, and diabetes) with variable cut-offs defined by Youden's index, the model predicting any hepatic fibrosis (Fibroscan LSM > 7 kPa) had an area under the curve (AUC) of 0.75 (95% CI 0.67–0.84), negative predictive value (NPV) of 91.5% and positive predictive values (PPV) of 40.0%. The equation includes three predictor variables: DM (yes/no), BMI (> 33 vs. ≤ 33), and FIB- 4 (> 1 vs. ≤ 1).

We will apply our e-trigger [7] on updated data extracted from the electronic health records, which include demographics, diabetes mellitus (DM) status, body mass index (BMI), alanine transaminase (ALT), aspartame aminotransferase (AST), and platelet count (PLT) levels within the past 12 months. FIB- 4 will be calculated based on ALT, AST, PLT, and age (at time of laboratory test completion) using the formula [FIB- 4 = age (years) × AST (U/L)/[platelet counts (10^9^/L) × sqrt(ALT) (U/L)]. DM presence is defined as having at least one DM-related prescription filled and dispensed within the last year.

Patients who have all three conditions above (DM + high BMI [> 33] + high FIB- 4 [> 1]) are considered as e-trigger positive patients. Lists of e-trigger positive patients with their e-trigger data will be made available to the PACT leads and/or nurse who will perform the rest of the steps after receiving the study’s education and training.

To ensure that patients are active and have the opportunity to meet with their provider regarding their health condition, we will generate e-trigger data for patients scheduled for a visit within the next three months. Phone call visits will be excluded as they are typically brief and primarily address lab results or medication changes.

**Fig. 4:**
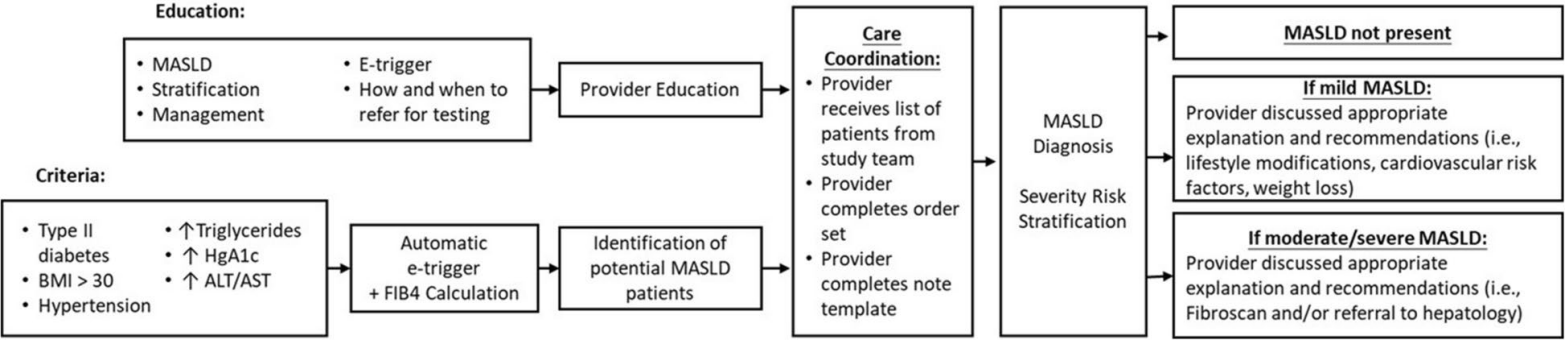
MASLD intervention

The education component of our intervention will last 2–3 weeks and will include structured and mostly didactic information on MASLD diagnosis and management, the e-trigger, and referring/managing patients through the MCCP. Training will be based on adult learning theory principles in an interactive presentation led by the study team that will include a power point presentation, role play, structured discussion and open discussion (Fig. 4).

### Usual care PACTs

We will provide PACT members in the usual care study arm with published MASLD practice guidelines [7] and MASLD patient education material (identical to that provided to the MCCP PACTs) but without the e-trigger information or the detailed educational and training component. Patients in the usual care arm will receive standard of care for diagnosis and management of MASLD.

### Outcomes

Patient-level clinical outcomes will be measured at six and 12 months following the delivery of the e-trigger results to the PACT. The primary outcome will be a composite dichotomous variable of making a new MASLD diagnosis and completing severity risk stratification of MASLD. Secondary outcomes include referral and receipt of hepatology specialty care for patients with MASLD and advanced fibrosis.

### Data collection

#### Focus groups with healthcare professionals and patient interviews

For formative assessment in Aim 1, we will conduct focus groups with six PACT team members including PCP, nurse care manager, clinical associate and administrative clerk to assess feasibility and acceptability of MCCP in primary care. We will also conduct 20 individual patient interviews to explore patient perspectives on the proposed intervention.

#### Electronic data extraction and chart reviews

For most measures, we will use automated structured data such as ICD codes, lab values, pharmacy and imaging in the VA electronic health records (Table 1). We will use chart reviews to extract data regarding the primary outcome measure, existence of electronic health record (EHR) progress note documenting a new MASLD diagnosis and complete risk stratification (e.g., fibrosis).

**Table 1:** Data sources

Prognostic Measures	Measures/Sources	Baseline	6 Months	12 Months
Demographics	CDW	✓		
Medications (liver and non-liver)	CDW	✓		
Comorbidities	CDW	✓		
VA healthcare utilization	CDW	✓		
Adherence to ambulatory care	CDW	✓		
Outcomes				
MASLD detected and risk stratified*	CDW/EHR	✓	✓	
Testing with Fibroscan	CDW	✓	✓	✓
Referral for hepatology	CDW consult table	✓	✓	✓
Seen in hepatology clinic	CDW	✓	✓	✓
Documentation of MASLD discussion	Chart review		✓	✓
Referral to weight loss services	CDW consult table	✓	✓	✓
Enrollment in weight loss services	CDW	✓	✓	✓

*CDW* corporate data warehouse, *EHR* electronic health record

*primary outcome measure

#### Interviews with healthcare professionals and patients

For summative evaluation in Aim 3, we will conduct interviews with six PCPs and six nurses in the MCCP arm and three PCPs and three nurses in the usual care arm. These interviews will query aspects of adoption and implementation, such as barriers and facilitators to the referral process and adaptations made while delivering the intervention.

#### Data quality and storage

We will verify the study outcome using data from the *Compensation and Pension Record Interchange (CAPRI)*, which provides access to all VA text notes, including progress notes, pathology, and imaging reports. We have used CAPRI for several studies, including to examine the validity of NAFLD diagnosis, NAFLD care, and cirrhosis diagnosis and care. Personal and health information about study participants will be securely stored on secure servers and/or locked cabinets before, during, and after the study in accordance with institutional and IRB guidelines.

### Sample size

Over the study period, we anticipate including ~ 8000 patients in the intervention arm and ~ 8000 in the usual care control arm. We estimated sample size and power for the parallel, cluster-randomized trial based on the number of clusters (8 per arm) to detect the anticipated effect size in the percentage of MASLD detected and risk stratified (7% for usual care vs. 17% for MCCP) with the intracluster correlations coefficients (ICC) as large as 0.04. To account for dependency among patients within a PACT, the design effect was applied following the approach of Killip et al. [25]. Applying this adjustment, with 16 total PACTS there is 84% to 99% power to detect a difference of 7% versus 17% with ICCs ranging from 0.02 to 0.04. In the event PACT attrition in the MCCP group is as high as 2 (attrition is not possible in the usual care arm), there is 80% to 99% power to detect desired effects with ICCs 0.02 to 0.04.

### Data analysis

#### Analysis plan for formative assessment

Qualitative data from PACT focus groups and patient interviews will be analyzed using rapid analysis [26] to allow the research team to move through qualitative data analysis in a timely manner to produce rigorous findings that will inform MCCP intervention adaptation. The qualitative methodologist will summarize transcripts using a template designating content into CFIR constructs. Summaries will identify emergent themes and key insights from interviews to be captured and documented. For focus group summaries, the role of the different providers will be noted alongside text to track source and allow analysts to consider how position could influence perspective. We will use matrixes to explore similarities, differences and patterns in experiences with screening and MASLD care across PACT teams and patients.

#### Analysis plan for cluster-randomized trial

We will compare baseline outcome variables for demographics (e.g., age, gender, race), medications (e.g., diabetes, dyslipidemia, hypertension), comorbidities (i.e. metabolic conditions, other medical or mental health conditions), healthcare utilization (e.g., outpatient, inpatient encounters), and adherence to ambulatory care for the MCCP versus usual care participants using SAS Proc Mixed for continuous variables and SAS Proc Glimmix for categorical variables. We will also compare baseline PACT characteristics (e.g. number of clinician and provider mix) between the study groups. Variables that differ between the study groups will be included as covariates in subsequent outcome analyses. Outcome analyses will be performed at 6- and 12-months post-enrollment for both primary (i.e. dichotomous composite of a new MASLD diagnosis and risk stratification) and secondary (i.e. testing with Fibroscan, referral for hepatology/weight loss services) outcomes. Intention -to-Treat and Per-Protocol analyses will employ multilevel models that account for dependency of patients within PACTs. SAS Proc Glimmix will be used for each dichotomous outcome with study group (MCCP or usual care) as the main predictor and variables that differ between study group at baseline will be included as covariates.

#### Mixed-methods approach for summative evaluation

Based on the RE-AIM framework, we will use a mixed-methods approach with qualitative and quantitative data to summatively evaluate the MCCP (Table 2). For example, *reach* will be assessed quantitatively by comparing patient characteristics for patients who are referred to weight loss (i.e., exercise program, endocrine clinic, medications, endoscopic or surgical bariatric procedures) and/or hepatology within a given intervention PACT and those who are not within the same PACT. For *implementation*, we will use descriptive statistics to examine the average degree of fidelity to the intervention at the PACT level. We will also examine the association between the degree of fidelity and each binary outcome at 6 and 12 months.

**Table 2:** RE-AIM elements and corresponding measures

RE-AIM Element	Corresponding Measure
Reach: *proportion and representativeness of individuals who participate in an intervention*	1) Characteristics of MASLD patients who are referred to weight loss in a given PACT compared with characteristics of all MASLD patients in a given PACT* 2) Characteristics of MASLD patients who are referred to hepatology in a given PACT compared with characteristics of all MASLD patients in a given PACT* 3) Differences between the intervention and control groups in the relationship between patient characteristics and each primary and secondary outcome at 6 months (see Effectiveness)
Effectiveness: *impact of an intervention on important outcomes*	1) Differences in the proportions of patients with MASLD diagnosis and risk stratification between intervention and control groups* 2) Differences in the proportions of patients with MASLD who are referred to a weight loss program within 6 months between intervention and control groups * 3) Differences in the proportions of patients with advanced MASLD who are referred to hepatology within 6 months between intervention and control groups* 4) Differences in the proportion of patients with MASLD who are referred to a weight loss program and start the program within 6 months between intervention and control groups* 5) Differences in the proportion of patients with advanced MASLD referred to hepatology and are seen in a hepatology clinic within 6 months between intervention and control groups*
Adoption: *proportion and representativeness of individuals who deliver the intervention*	1) Characteristics of PACTs participating in the study (make up of clinicians, number of clinicians in PACT) and differences between intervention and control groups in the relationship between PACT characteristics and each outcome at 6 months* 2) Differences among PACTs in frequency of referrals to weight loss and hepatology** 3) Barriers and facilitators to referral to weight loss and hepatology**
Implementation: *fidelity to elements of intervention*	1) Fidelity to MCCP intervention in terms of education/referral patterns and the relationship between degree of fidelity and each outcome at 6 months (based on documentation of checklist in CPRS)* 2) Adaptations of MCCP delivery by PACT members** 3) Time required to deliver MCCP as reported in interviews** 4) Patient experiences with PACT and providers using MCCP**
Maintenance: *extent to which intervention effects are sustained 6 or more months* (Examination of outcomes at 12 months)	1) Differences in the proportion of patients with MASLD who are NEWLY referred to weight loss program (within 6–12 months) between intervention and control groups* 2) Differences in the proportions of patients with advanced MASLD who are NEWLY referred to hepatology (6–12 months) between intervention and control groups* 3) Differences in the proportion of patients with MASLD who are referred to a weight loss program who actually start the program within 12 months between intervention and control groups 4) Differences in the proportion of patients with advanced MASLD referred to hepatology who actually see a hepatology clinic within 12 months between intervention and control groups

*quantitative measure

**qualitative measure

Qualitative data will include interviews with PCPs and nurses in both study groups to explore barriers and facilitators to the referral process and adaptations made while delivering the intervention (Table 3). Semi-directed content analysis will be used to analyze interview data [27]. This approach leverages pre-determined themes to structure analysis and allows new themes to emerge. RE-AIM framework will inform parent codes (e.g., adoption, implementation). Sub-codes (e.g., adoption: decision making) will allow for granular analysis and capture of various experiences.

**Table 3:** PACT focus group questions for summative evaluation

Adoption
• *Intervention-arm:* Please share your initial perspective on MCCP. Did you have any specific concerns? Was there anyone in your PACT that seemed reluctant/resistant to any/all of the MASLD CCP intervention? • *Control arm:* Please share your initial perspective on MCCP. Did you have any specific concerns? • *Intervention-Arm:* About how long did it take to go from receiving notification about a patient at-risk for MASLD and referring them to weight loss/hepatology? What were some challenges encountered throughout this process?
Implementation
• What challenges were associated with the MCCP training? • About how long did it take to counsel/educate patients at-risk for MASLD? If/how did this impact the flow of the clinic? What were some challenges encountered throughout this process?

## Discussion

Our intervention addresses an important gap in clinical care that affects a large and steadily growing number of patients with MASLD especially in the primary care setting. While our intervention is based on a sound conceptual framework, rigorous testing for effectiveness and eventually implementation is still required. Primary care providers are facing a growing list of tasks, and their prioritization is at least partly dependent on the frequency of conditions, its impact, the evidence underlying the intervention and the availability of downstream management options. Furthermore, high level evidence is required for professional societies, medical practices and payors to accept and adopt potentially expensive interventions.

There are multiple factors that may influence the effectiveness of the intervention. First, the performance characteristics of the e-trigger in correctly detecting patients with high likelihood of having significant MASLD and excluding patients without this condition. Our intervention incorporates risk stratification at multiple steps. The performance of non-invasive tests (e.g., FIB- 4, Fibroscan) is considerably improved when applied to groups with high pretest likelihood of having MASLD or MASH. Second, the responsiveness and reactions from health care providers when faced with patients flagged for high likelihood of MASLD. Third, the feasibility and effectiveness of downstream measures (e.g., obtaining further testing or referrals). Last, we need to demonstrate the favorable change in objective outcomes in relation to the e-trigger. While long-term MASH outcomes such as development of cirrhosis, hepatocellular carcinoma, liver failure or mortality are considered gold standard, within a feasible follow up our trial will focus on intermediate outcomes (correct diagnosis and risk stratification, referral for weight loss and or specialty care) that are likely to correlate well with the long term outcomes.

The proposed trial has several strengths. The algorithm being tested provides a fundamental paradigm shift in MASLD care from haphazard, sporadic, and mostly guideline unconcordant care into a systematic, structured, equitable, and evidence-based approach. Information on the effectiveness of the clinical care pathway for screening, risk stratification, and management is also essential for healthcare planning and resource allocation. The study uses the rigorous approach of cluster-randomized controlled trial design and incorporates pragmatic features that facilitate subsequent implementation. The sampling frame for randomization is at the level of PACTs in primary care, which will reduce contamination between randomized groups. However, outcomes are at the individual level because improved patient care is the ultimate goal. The study utilizes well-validated techniques to non-invasively diagnose MASLD, and stage the severity of hepatic fibrosis. The focus on the primary care setting is relevant because this is where most delays in MASLD diagnosis occur. Our summative evaluation aim will provide key information about the determinants of feasibility, acceptability, and implementation of data-driven derived clinical prediction rules and pathways.

The proposed study is notable in that it includes strategies from implementation science during an effectiveness trial. Frequently, implementation science methods are not included in effectiveness studies and are employed in hybrid studies until after effectiveness has been established. By including implementation science methods early in this effectiveness trial, we are able to balance a rigorous study design and assess the feasibility and relevance of implementing an e-trigger in primary care. This approach will generate timely, real-world findings to facilitate implementation of the MASLD e-trigger into practice locally and nationally.

The study also has limitations. The availability, quality, and nature of data contained with electronic databases could limit the accuracy of MCCP; however, all e-triggers ultimately involve manual review of trigger-positive records. FIB- 4 calculation will not be possible initially in 10–15% of patients but the relevant labs (AST, ALT, platelets) will be requested as part of the scheduled outpatient visit; therefore, we expect the completion of this step in > 95% of cases. A few patients (< 10%) could fail the Fibroscan test by having no or non-meaningful reads. For these, the hepatology service will request MR elastography. Lastly, the generalizability of the findings to other practice settings may not be possible, although all the elements of the study are widely available.

In conclusion, the MCCP study will provide high-quality data to inform the diagnosis and management of a common condition in primary care. Our trial will be first to systematically address the MCCP using strategies that are Veteran-centric, clinically relevant, adaptable and scalable. If successful, the MCCP will improve the detection, risk stratification, and early treatment of patients with MASLD.

## Supplementary Information

Supplementary Material 1. 

## Data Availability

No datasets were generated or analysed during the current study.

## Abbreviations

ALT: Alanine transaminase
AST: Aspartate aminotransferase
AUC: Area under the curve
BMI: Body mass index
CDW: Corporate data warehouse
CFIR: Consolidated Framework for Implementation Research
DM: Diabetes mellitus
EHR: Electronic health record
ICC: Intracluster correlations coefficients
MASH: Metabolic dysfunction associated steatohepatitis
MASLD: Metabolic dysfunction associated steatotic liver disease
MCCP: MASLD clinical care pathway
MEDVAMC: Michael E. DeBakey VA Medical Center
NPV: Negative predictive value
PACTs: Patient Aligned Care Teams
PLT: Platelet count
PPV: Positive predictive value
RE-AIM: Reach, Effectiveness, Adoption, Implementation and Maintenance
SELFIE: Sustainable intEgrated care modeLs for multi-morbidity: delivery, Financing and perfromanceE
VA: Veterans Affairs
